# MicroRNA miR-451 downregulates the PI3K/AKT pathway through CAB39 in human glioma

**DOI:** 10.3892/ijo.2011.1306

**Published:** 2011-12-15

**Authors:** YUAN TIAN, YANG NAN, LEI HAN, ANLING ZHANG, GUANGXIU WANG, ZHIFAN JIA, JIANWEI HAO, PEIYU PU, YUE ZHONG, CHUNSHENG KANG

**Affiliations:** 1 Department of Neurosurgery, Tianjin Medical University General Hospital, Tianjin 300052, P.R. China; 2 Laboratory of Neuro-Oncology, Tianjin Neurological Institute, Tianjin 300052, P.R. China; 3 Key Laboratory of Neurotrauma, Variation and Regeneration, Ministry of Education and Tianjin Municipal Government, Tianjin 300052, P.R. China

**Keywords:** glioma, microRNA-451, target gene, calcium binding protein 39

## Abstract

The microRNA miR-451 is downregulated in gliomas, this has been suggested by several different research groups and is consistent with our data. Our previous study also confirmed that miR-451 has a repressive role in glioma by inhibiting cell growth, proliferation and by inducing cell apoptosis. In the present study, we identified a target gene of miR-451 in human glioma and investigated the mechanism for the glioma suppressive effect of miR-451 functions. Expression of miR-451 in gliomas was identified by quantitative real-time PCR and fluorescence *in situ* hybridization. Human glioma cell lines (U251, U87, LN229 and A172) were transfected with miR-451 mimics to restore miR-451 expression. The tumor suppressive effects of miR-451 were further verified by subcutaneous assays in nude mice, in addition to our previous *in vitro* data. A candidate target gene was tested by Western blotting and luciferase reporter assays. Some PI3K/AKT pathway factors were tested by Western blotting. We found that miR-451 expression was downregulated in glioma samples and was inversely correlated with WHO grades of gliomas. *In vivo* assays confirmed that miR-451 had tumor suppressive traits. CAB39-3′UTR luciferase reporter assay confirmed CAB39 as a direct target gene of miR-451. Significant alterations in the expression of PI3K/AKT pathway factors were observed by Western blot assays. We conclude that miR-451 represses glioma *in vitro* and *in vivo*, likely through targeting CAB39 directly and inhibiting the PI3K/AKT pathway indirectly.

## Introduction

Glioblastoma multiforme (GBM) is the most common, lethal and aggressive type of primary brain tumor with a median survival of 9–12 months. Despite advances in the basic understanding of cancer biology and therapeutic advances in other neoplasms, the poor prognosis for GBM has not improved in the last four decades and its treatment remains ineffective and essentially palliative (1,2). Though the exact molecular mechanisms for glioma genesis remain unclear, recent studies have reported that there were several microRNA (miRNA or miR) abnormalities in human gliomas, including miR-451 (3). miRNAs are now being used as a new type of molecules of interest to elucidate tumorigenesis and novel therapeutic approaches could be developed by targeting miRNAs that are altered in glioma (4).

miRNAs are a class of small (~22 nucleotides) non-coding RNAs that function as negative regulators of gene expression at the post-transcriptional level by binding to complimentary sequences in, mainly, the 3′-untranslated regions (3′-UTRs) of specific mRNAs (5–11). These miRNAs play important roles in apoptosis, proliferation, differentiation, development, and metabolism (12–14). In particular, miRNAs show altered expression in tumors in relation to normal tissues and miRNA aberrations may be important in tumor progression (15). hsa-miR-451 is located on chromosome 17q11.2, a region known to be amplified in certain types of cancers, in close proximity to ERBB2 (17q12) (16,17). Studies have shown that miR-451 inhibited cell growth (3), proliferation, and invasion and enhance apoptosis (18). The identification of target genes associated with altered miRNA expression might accurately elucidate the role of miRNAs in cancer biology (15).

In this study, we confirmed the hypo-expression of miR-451 in gliomas using quantitative real-time PCR and fluorescent *in situ* hybridization. The calcium binding protein 39 gene (CAB39) predicted by bioinformatics analysis as a target gene of the miR-451, was validated by fluorescent reporter assay. We further analyzed the signaling pathway miR-451 might regulate in human glioma and found that miR-451 modulated the expression of multiple downstream molecules such as LKB1, AMPK, PI3K and AKT, suggesting that miR-451 may act as a tumor-suppressor factor and regulate the PI3K/AKT pathway through LKB1 and AMPK.

## Materials and methods

### Patients and samples

Tissue specimens and clinical information were obtained as part of an approved study by the Institutional Review Board at the Tianjin Medical University, China. Forty-six human glioma tissues were collected with patient consent at the time of operation, grading of tumors was carried out with WHO criteria (World Health Organization, 2007). The tissue samples included: 3 grade I tumors (3 hairy cell astrocytoma); 8 grade II tumors (1 protoplasmic astrocytomas, 6 fibrocytic astrocytomas and 1 mixed oligoastrocytomas); 15 grade III gliomas (all of these tumors were anaplastic astrocytomas); and 15 grade IV glioblastomas (GBMs). Five normal brain tissues were obtained from patients with traumatic brain injury and brain tumors for internal decompression. Immediately after surgery, samples were snap-frozen and stored in liquid nitrogen.

A glioma tissue microarray was purchased from Shaanxi Chaoying Biotechnology (Xi’an, China). Pathologic grades of tumors on the microarray were defined according to the 2007 WHO criteria as follows: 4 grade I tumors (4 Pilocytic astrocytoma), 18 grade II tumors (15 astrocytoma, 2 oligoastrocytoma and 1 oligodendroglioma), 14 grade III tumors (10 anaplastic astrocytoma, 3 anaplastic oligoastrocytoma and 1 anaplastic oligodendroglioma), 39 grade IV tumors (all glioblastomas); 5 were normal brain tissue. Each dot represented a tissue spot from one individual specimen, selected and pathologically confirmed. The array dot diameter was 1.5 mm. All microarrays were stored in the dark at 4°C.

### In situ hybridization

Using sense locked nucleic acid (LNA)-modified oligonucleotide probes, *in situ* hybridization was performed with an *in situ* hybridization kit (Boster Biological Technology, Ltd., Wuhan, China). The sequences of the LNA/DNA oligonucleotides contained locked nucleic acids at five consecutive centrally located bases (indicated by the underline) as shown: HSA-miR-451 5′-TTGAG TCATT ACCAT TGCCA AA-3′. The glioma tissue microarrays were deproteinated, and then prehybridized for 2 h in hybridization liquid in a humidified chamber (50% formamide, 5 × SSC). The probes (miR-451 10 ng) were added to the sections on the microarray and incubated overnight at 40°C in a water bath. After washing with PBS 3 times, the same volume of anti-digoxigenin-rhodamine and streptavidin-FITC solution was added and incubated for 2 h at room temperature in the dark. Nuclei were counterstained with a DAPI karyotyping kit (Genmed, USA). After washing with PBS 3 times, sections were sealed and detected under a fluorescence microscope with an OptiGrid system and analyzed by IPP6.1 (Olympus, Tokyo, Japan).

### Analysis of hsa-miR-451 candidate target genes

**Figure 1:**
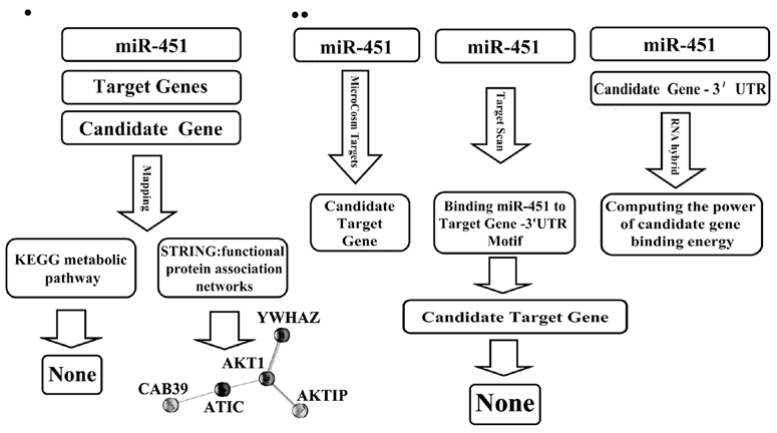
Pipeline for the analysis of the hsa-miR-451 candidate target genes using miRNA target prediction software (TargetScan and MicroCosm Targets), STRING, the functional protein association network database, the KEGG pathway database and RNAhybrid.

Previous studies in our laboratory have shown a negative correlation between miR-451 and the expressions of AKT1 and c-Myc (18). We further analyzed our data using several databases (Fig. 1). First, we used TargetScan 5.1 (http://www.targetscan.org) to search for candidate miR-451 target genes and found 14 (YTHDF2, ZNF644, CUGBP2, C11orf30, FMNL3, FBXO33, AKTIP, VAPA, RKHD2, SAMD4B, OSR1, TTN, CAB39, YWHAZ). Next, we used STRING, the functional protein association networks database (http://www.bork.embl-heidelberg.de/STRING/) (19), to explore possible interactions between the 14 candidate miR-451 target genes and AKT1 and c-Myc. To exploit the possible interactions that were identified between the miR-451 target genes and AKT1 and c-Myc we used the KEGG pathway database (http://www.genome.jp/kegg/) (20). For a second prediction of possible hsa-miR-451 (human miR-451) gene targets we used MicroCosm Targets (http://microrna.sanger.ac.uk/) (21) and investigated whether AKT1 and c-Myc were included in the prediction. Finally, to further evaluate the possibility of AKT1 and c-Myc as hsa-miR-451 target genes we used RNAhybrid (http://bibiserv.techfak.uni-bielefeld.de/rnahybrid) (22).

### Cell culture and transfection

The human GBM cell lines LN229, U87 and U251 were purchased from the Institute of Biochemistry and Cell Biology, Chinese Academy of Sciences, Shanghai, China. The human glioblastoma cell line A172 was gifted by Professor Jinhuan Wang (Tianjin First Central Hospital, China). The cells were maintained in Dulbecco’s modified Eagle’s medium (DMEM, Gibco, USA) supplemented with 12% fetal bovine serum (Invitrogen, Carlsbad, USA), and incubated at 37°C with 5% CO_2_. Transfections with hsa-miR-451 mimics were performed in serum-free medium 24 h after plating, with Lipofectamine 2000 (Invitrogen). The oligonucleotide sequence of the hsa-miR-451 mimics was: 5′-AAACCGUUACCAUUACUGAGUU-3′. A scrambled siRNA sequence (5′-TTCTCCGAACGTGTCACGT-3′) was used as the negative control (Gima Biol Engineering Inc., Shanghai, China). Cells were then cultured in complete medium 6 h later.

### Quantative real-time PCR analysis

Total RNA of the GBM cells (U251, LN229, A172, U87), as well as of the 46 snap-frozen human glioma tissues was harvested using TRIzol (Invitrogen) following the manufacturer’s protocol. The concentration and purity of RNA were determined using NanoDrop^®^ ND-1000. Total RNA (2 μg) was used for cDNA synthesis by reverse transcription using M-MLV Reverse Transcriptase (Promega) according to the manufacturer’s protocol. Expressions of mature miR-451 were quantified by miR-qRT PCR using the Hairpin-it™ miRNA qPCR Quantitation kit (GenePharma Co. Ltd.). All PCR reactions were performed using standard PCR conditions: stage 1: 95°C for 3 min (1 cycle); stage 2: 95°C for 12 sec, followed by 62°C for 40 sec; stage 3: from 62°C up to 95°C, followed by 0.2°C for 2 sec (1 cycle). U6 was used as the internal control. Data are shown as fold change and analyzed initially using Opticon Monitor Analysis Software V2.02 software (MJ Research, Waltham, MA, USA).

### Luciferase activity assay

The pGL3-CAB39-3′UTR-Subcloning and pGL3-CAB39-3′UTR-Mut plasmids were purchased from GenScript (Nanjing, China). The cDNA was cloned into the *Xba*I/*Xba*I site of the pGL3-control vector downstream of the luciferase gene, to generate pGL3-CAB39 vectors with the following oligonucleotide sequences (Fig. 3C): The gene CAB39 3′UTR-Wild: 3′-TCTAGATGTTAGCTATTCAG CATCAGGCACTCTTATTGATTCATGAGGAACACTGC TAATCTGCTGTTAAGTGAACGGTTTTTCATTTTACCCT TTTGTTTTTCAGTCCAGGTTGGAGATCGTAGCTGCTG CTGCTTGCACACTCTAGAA-5′; The gene CAB39 3′UTR-Mut: 3′-TGTTAGCTATTCAGCATCAGGCACTCTTATTGA TTCATGAGGAACATTACTGCTAATCTGCTGTTAAGTGC CATTGGGTTCATTTTACCCTTTTGTTTTTCAGTCCAGG TTGGAGATCGTAGCTGCTGCTGCTTGCACACC-5′.

For the luciferase reporter assay, the U251, LN229, U87 and A172 cells were cultured in 96-well plates (2000 cells per well), transfected with 5 pmol of the hsa-miR-451 mimic oligonucleotide with Lipofectamine 2000. 24 h after transfection, the cells were transfected again, this time with 0.2 μg of either the pGL3-CAB39-3-UTR-Subcloning plasmids or the pGL3-CAB39-3′UTR-Mut plasmids with Lipofectamine 2000. After 48 h of this transfection, luciferase activity was measured using the Luciferase Assay System (Promega).

### Western blot analysis

Cells were harvested 48 h after transfection, rinsed three times with ice-cold PBS and then extracted with 1% Nonidet P-40 lysis buffer (20 mM Tris, pH 8.0, 137 mM NaCl, 1% Nonidet P-40, 10% glycerol, 1 mM CaCl_2_, 1 mM MgCl_2_, 1 mM phenylmethylsulfonyl fluoride, 1 mM sodium fluoride, 1 mM sodium orthovanadate, and a protease inhibitor mixture). Homogenates were clarified by centrifugation at 20,000 × g for 15 min at 4°C, and protein concentrations were measured by Nanodrop spectrophotometer (Gene, USA). Protein lysates (50 μg) from each sample were subjected to sodium dodecyl sulfate-polyacrylamide gel electrophoresis (SDS-PAGE) on 10% SDS polyacrylamide gel. The separated proteins were transferred to polyvinylidene difluoride (PVDF) membranes (Millipore, Billerica, MA). The membrane was incubated with primary antibodies against CAB39, LKB1, AMPK, p-AMPK, PI3K (p110α) and p-AKT1/2/3 (1:1000 dilution, Santa Cruz, USA), followed by incubation with an HRP-conjugated secondary antibody (1:1000 dilution, Zhongshan Bio Corp, Beijing, China). The specific protein was detected using a SuperSignal protein detection kit (Pierce, USA). After washing with stripping buffer, the membrane was re-probed with antibody against GAPDH (1:1000 dilution, Santa Cruz).

### Subcutaneous tumor assay

BALB/c-A 6-week-old nude mice were purchased from the animal center of the Cancer Institute of Chinese Academy of Medical Science and housed individually in ventilated microisolator cages with water and food. All experimental procedures were carried out according to the regulations and internal biosafety and bioethics guidelines of Tianjin Medical University and the Tianjin Municipal Science and Technology Commission. The LN229 subcutaneous tumor xenograft model was established as previously described (23). When the tumors were approximately 5 mm in length, the mice were randomly divided into 3 groups (10 mice per group): the LN229 control group, the LN229 scramble PBS-treated group, and the LN229 miR-451-treated group. A mixture of 5 μl oligonucleotides containing scramble miR-451 mimics and 10 μl Lipofectamine was injected into the xenograft tumor model in a multi-site injection manner. The mice in the LN229 control group received 10 μl of PBS only. Treatment was conducted every four days, until the end of the experiment. The tumor volume was measured with a caliper every 3 days using the formula, volume = length × width^2^/2. At the end of a 21-day observation period, the mice were sacrificed and the tumor tissues were removed for formalin fixation and preparation of paraffin-embedded sections for immunohistochemical analysis.

### Immunohistochemistry analysis

The paraffin-embedded tissue sections were examined for CAB39, AMPK, p-AMPK, LKB1, PI3K, p-AKT, and GAPDH expression, while the glioma tissue microarrays were examined for CAB39 expression, and H&E staining. Sections were dewaxed, treated with 3% H_2_O_2_ for 10 min, and incubated with appropriate primary antibodies (1:100; Santa Cruz Biotechnology) overnight at 4°C. They were then treated with biotinylated secondary antibody (1:100) for 1 h at room temperature, followed by incubation with avidin-biotin complex (ABC)-peroxidase for a further 1 h. After washing with Tris buffer, the sections were incubated with 3,3′-diaminobenzidine (DAB) for 5 min, rinsed in water, counterstained with hematoxylin, and visualized using a light microscope.

### Statistical analysis

SPSS10.0 was used for statistical analysis. One-way analysis of variance (ANOVA) and χ^2^ test was used to analyze the significance between groups. The LSD method of multiple comparisons with parental and control vector groups was used when the probability for ANOVA was statistically significant. Statistical significance was determined at p<0.05.

## Results

### miR-451 expression was negatively correlated with the WHO grades of gliomas

**Figure 2:**
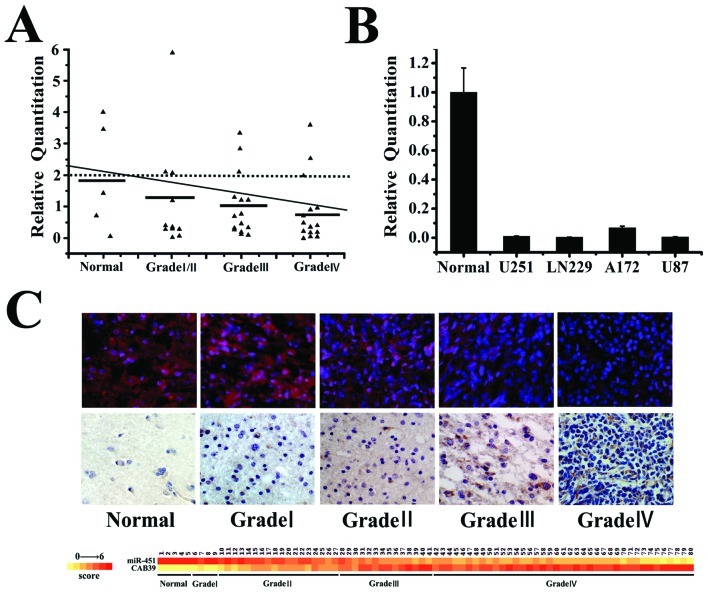
The *in vitro* and *in vivo* relationships between miR-451 expression and glioma grades. (A and B) The correlation between miR-451 expression and glioma WHO grades by qRT-PCR. (C) The correlation between miR-451 expression and glioma WHO grades by *in situ* hybridization. (A and C) Normal brain tissues and gliomas of different WHO grades were compared; (B) normal brain tissues and four different glioblastoma cell lines were compared.

To further study the biological role of miR-451 in human glioma tissues, we examined miR-451 expression in normal brain tissues, glioma tissues and glioma cell lines by quantitative RT-PCR. As shown (Fig. 2A and B), miR-451 expression decreased with the increasing WHO grades of glioma tissues. *In situ* hybridization analysis revealed that miR-451 was expressed in gliomas and its total positive rate was 94.67% (71/75). The expression of miR-451 decreased markedly in high grade gliomas (WHO grades III and IV) compared to its expression in low grade gliomas (WHO grades I and II). Indeed, 22/22 low grade gliomas exhibited detectable levels of miR-451, while in 4/53 high grade gliomas miR-451 was at detectable levels (p<0.05) (Fig. 2C).

### CAB39 is a target gene of miR-451 in glioma

Although the reported under-expression of miR-451 in some types of tumors suggested a role in cancer development, the underlying mechanism is still unclear because little is known about the miR-451 target genes. Therefore, the identification of miR-451-regulated targets is a necessary step to understand how miR-451 functions. We used a three-step consequential approach to identify miR-451 target genes. First, target genes were predicted by bioinformatics analysis, then, predicted genes were tested with Western blotting and finally, potential target genes were validated by fluorescent reporter assay (25).

### miR-451 target gene(s) identification

Bioinformatics analysis failed to identify AKT1 and/or c-Myc as hsa-miR-451 target genes. However, using the STRING proteins functional association network database, AKT1 was shown to have a direct association with AKTIP and YWHAZ and an indirect association with CAB39, all three of these genes were predicted to be hsa-miR-451 target genes (Fig. 1). CAB39-AMPK-mTOR-AKT1 was inferred in KEGG pathway database, with CAB39-AMPK-mTOR documented while AKT1 not. It is possible that hsa-miR-451 interacts indirectly with AKT1, through its target genes. Both the STRING and KEGG databases indicated that CAB39 was the preferential candidate target gene of hsa-miR-451.

### miR-451 target gene confirmation

**Figure 3:**
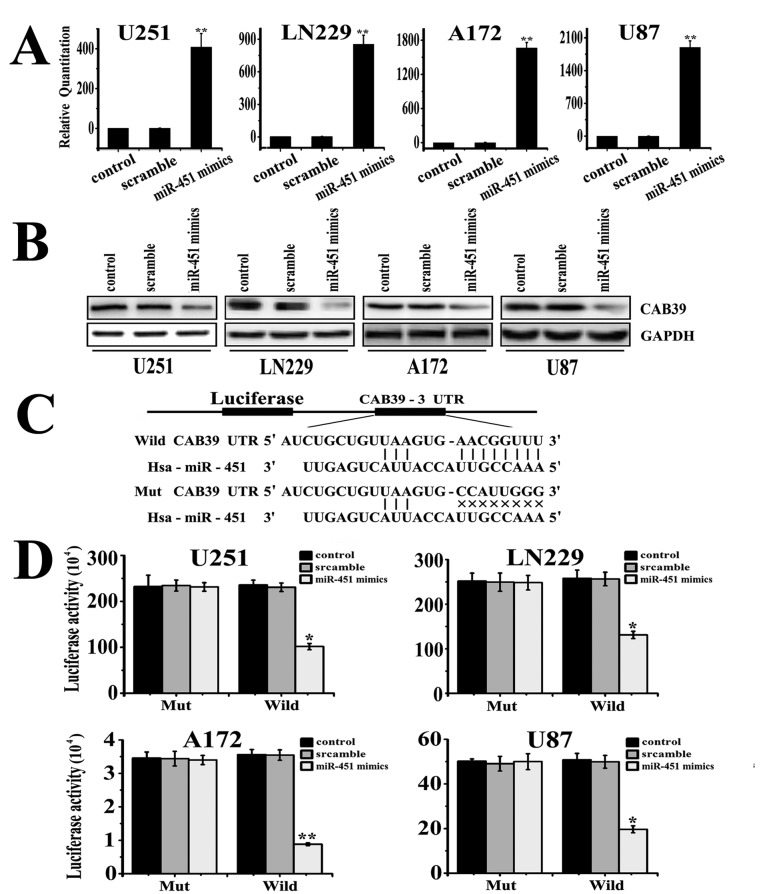
Analysis of CAB39 as a target gene of the miR-451. (A) Relative quantitation of CAB39, using quantitative real-time PCR, in U251, LN229, A172, and U87 cell lines following transfection with the miR-451 mimics (^*^p<0.05; ^**^p <0.01). (B) Western blot analysis showing CAB39 in the hsa-miR-451-mimics treated cells. (C) Schematic representation of the pGL3-CAB39 3′UTR containing reporter constructs. (D) Luciferase activity in the human glioma cells co-transfected with hsa-miR-451 and the pGL3-CAB39-3′UTR plasmids (p=0.0011).

Quantitative real-time PCR showed that miR-451 expression increased in U251, LN229, A172, and U87 cells by 340.14, 849.22, 1680.88 and 2033.85-fold respectively after transfection with the miR-451 mimics, compared to its expression in the control and scramble treated cells (Fig. 3A). The transfected cells were used in subsequent Western blot assays and CAB39 was found to be significantly downregulated in cells treated with the hsa-miR-451 mimic oligonucleotide. Finally, as shown in Fig. 3B, luciferase activity was significantly decreased in cells co-transfected with hsa-miR-451 and pGL3-CAB39-3′UTR-wild plasmid cells compared to its activity in the scramble, negative control and pGL3-CAB39-3′UTR-Mut plasmid-treated cells (p=0.0011, Fig. 3D). Together, these data demonstrated that CAB39 is a target gene of the miR-451 in glioma.

Previously we have shown that miR-451 expression was negatively correlated with glioma WHO grades in quantitative real-time PCR and *in situ* hybridization. Here, we found a similar phenomenon between CAB39 expression and glioma WHO grades (Fig. 2C), using immunohistochemistry. CAB39 was expressed in gliomas and its total positive rate was 96% (72/75). The levels of CAB39 increased markedly in high grade gliomas (WHO grades III and IV) in comparison to low grade gliomas (WHO grades I and II). Indeed, 53/53 high grade gliomas exhibited detectable levels of CAB39, while 3/22 low grade gliomas the protein was at undetectable levels (p<0.05).

### miR-451 regulated PI3K/ATK pathway factors in human glioma in vitro

**Figure 4:**
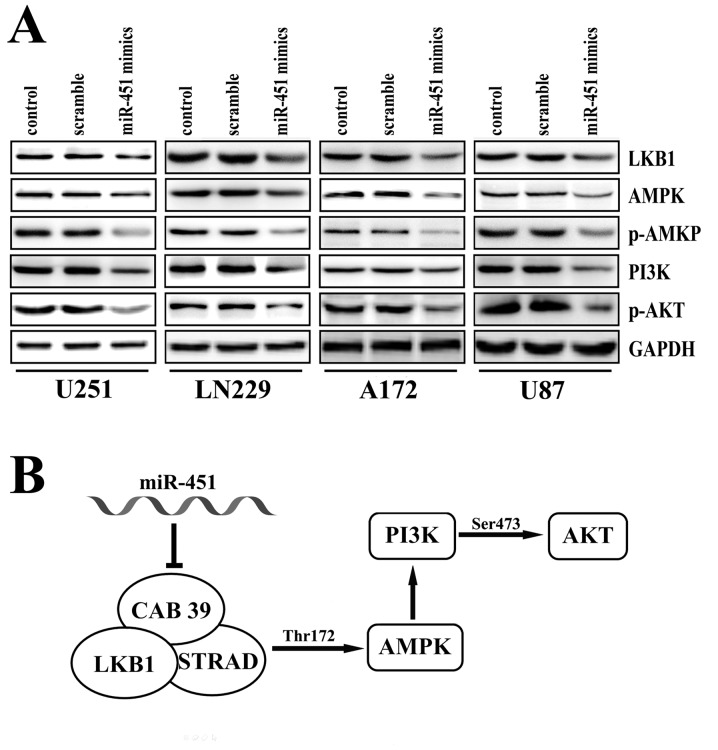
Impact of the miR-451 mimic oligonucleotide on some of the components of the PI3K/AKT signaling pathway. (A) U251, LN229, A172 and U87 cells were treated with the miR-451 mimics and LKB1, AMPK, p-AMPK, PI3K, p-AKT and GAPDH expressions were determined by Western blotting 48 h after treatment. (B) Schematic diagram of how miR-451 downregulation could lead to activation of the PI3K/AKT pathway.

Previous data from our laboratory showed that miR-451 had a significant impact on cell proliferation, invasion and apoptosis in human glioblastoma cell lines, possibly by regulating AKT expression (18). AKT1 plays a critical role in controlling a diversity of cellular functions, such as protein synthesis, cell cycle, cell survival and apoptosis (26,27). We, therefore, investigated AKT-related pathways. As shown in Fig. 4B, obvious activation of phosphorylated-AKT was observed in U251, LN229, A172 and U87 cells after transfection with the miR-451 mimics. Consistently, over-expression of miR-451 led to a marked down-regulation of LKB1, AMPK, p-AMPK, and PI3K, all of which are involved in the pathway upstream of AKT (Fig. 4A). These data suggest that the tumor suppressor activity of miR-451 in glioblastoma cells likely acts through its regulation of the PI3K/AKT pathway.

### miR-451 inhibited the growth of LN229 glioblastoma cells in vivo

**Figure 5:**
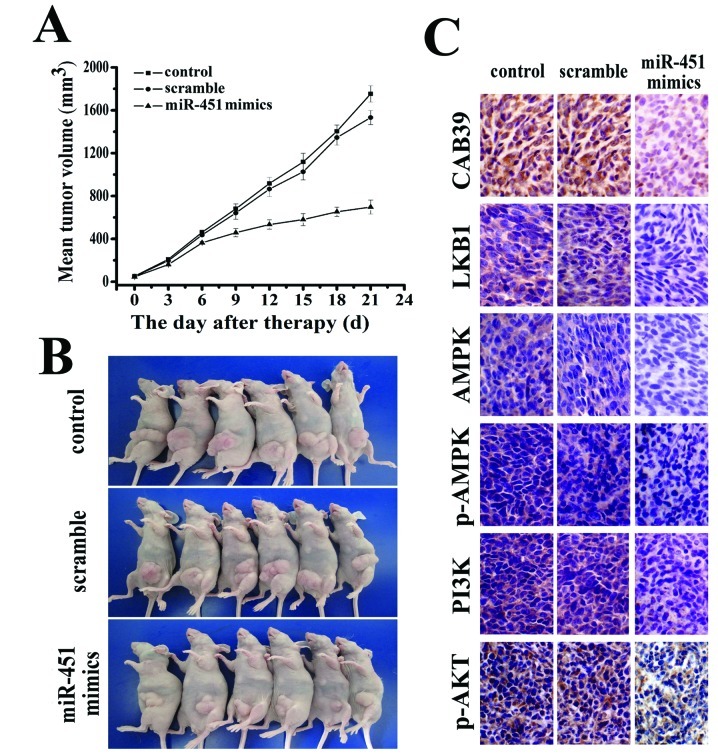
Effects of the upregulation of miR-451 on glioma growth *in vivo*. (A) *In vivo* comparison of mean tumor volume after treatment with the miR-451 mimics. (B) Representative animals from the 3 groups at the end of the study. (C) Immunohistochemistry analyses of xenograft tumors after treatment with the miR-451 mimics, comparing the expression levels of CAB39, LKB1, AMPK, p-AMPK, PI3K and p-AKT.

To further understand the anti-tumor effect of miR-451 and its role in the signaling pathway *in vivo*, we employed an LN229 xenograft glioma mouse model. The mean volume of the tumors used in this study prior to treatment was 56±20.35 mm^3^ (Fig. 5B). During the first 3 days of observation following intratumoral administration of miR-451, tumors in both the control and treated groups grew slowly with no marked differences in tumor size between them. Tumors in the group treated with miR-451 maintained a slow growth rate throughout the experiment while tumors in the control group began to grow faster. On day 12, tumors of the mice in the miR-451 treated group started to show statistically significant differences in size compared to tumors of the mice in the control group (p<0.05). At the termination of the study, the difference in tumor mass between the miR-451 treated group and the control group was marked (p<0.01). No difference in tumor volume was observed between the control and PBS-treated groups (Fig. 5A). To determine whether intratumoral miR-451 administration affected the expression of factors in the PI3K/AKT signaling pathway, the expressions of CAB39, LKB1, AMPK, p-AMPK, PI3K and p-AKT were tested and were found to show significant downregulation in an immunohistopathological examination (Fig. 5C).

## Discussion

miRNA expression profiling studies revealed that a number of miRNAs were dysregulated in human glioblastoma. miRNA-451 was one of them. A tumor suppressive role of miR-451 was shown in gliomas *in vitro* (18), but whether or not miR-451 actually participated in gliomagenesis still needs further investigation. Expression alteration can be a useful index of such activity. We used a high throughput experiment, microarray *in situ* hybridization examination, and further validated the result using the more quantitative and more sensitive quantitative real-time PCR test on clinical samples. miR-451 was down-regulated in glioma tissues and a significant negative correlation was revealed between miR-451 expression and glioma WHO grades.

Fluorescent reporter assay is generally accepted as a gold standard to determine miRNA targets. Using this assay, we demonstrated that CAB39 was a target gene of miR-451. CAB39 (MO25) is an armadillo repeat scaffolding-like protein with two isoforms, CAB39α and CAB39β (28,29). Structural studies have revealed that the CAB39α forms an extended α-helical repeat rod-like structure, distantly related to the armadillo repeat domain (30). CAB39 is a component of the trimeric LKB1-STRAD-MO25 complex (29,31) and its role is to stabilize the binding of STRAD to LKB1 and re-localize LKB1 from the nucleus to the cytoplasm (29,32). LKB1, a member of the serine/threonine kinase family, regulates cell polarity and functions as a tumor suppressor. Mutations in this widely expressed protein kinase in humans result in a disorder termed Peutz-Jeghers syndrome (PJS), which predisposes the sufferer to a wide spectrum of benign and malignant tumors (33,34). LKB1 is activated through its interaction with STRAD (37) and MO25 (29). AMP-activated protein kinase (AMPK) is a sensor of cellular energy charge which regulates physiological processes that consume or regenerate ATP to restore the energy charge in the cells (35). Studies in mammalian cells demonstrated that LKB1 complexed to STRAD and MO25 activated AMPK by phosphorylating Thr172, and that the STRAD and MO25 subunits enhanced phosphorylation of AMPK by over 100-fold (36,37). The phosphatidylinositol-3′ kinase (PI3K) family plays complex and extensive roles in many aspects of cell biology and metabolism (38). Signaling through PI3Ks is central to cell survival, growth and proliferation which, as a consequence, is frequently activated in many human cancers, including glioblastoma (39). Data from Luo *et al* provided new evidence that AMPK activated Akt by regulating PI3K (40).

To verify the significance of LKB1/AMPK, PI3K/AKT signaling in glioma, we performed Western blotting in human glioma cell lines and immunohistochemistry in a tumor xenograft mouse model. Consistently, over-expression of miR-451 led to a marked downregulation of factors upstream of AKT. This evidence, both *in vitro* and *in vivo*, implied that miR-451 can suppress cell proliferation in human glioma through the LKB1/AMPK and PI3K/AKT pathway. However, more evidence still needs to be found. Increasing the expression of miR-451 might be a useful therapeutic strategy for treating glioma in the future.
